# The Anticancer Drug Bleomycin Shows Potent Antifungal Activity by Altering Phospholipid Biosynthesis

**DOI:** 10.1128/spectrum.00862-22

**Published:** 2022-08-29

**Authors:** Mona Pokharel, Paulina Konarzewska, Jacques Y. Roberge, Gil-Soo Han, Yina Wang, George M. Carman, Chaoyang Xue

**Affiliations:** a Public Health Research Institute, New Jersey Medical School, Rutgers University, Newark, New Jersey, USA; b Molecular Design and Synthesis Core, Rutgers University Biomolecular Innovations Cores, Office for Research, Rutgers University, Piscataway, New Jersey, USA; c Rutgers Center for Lipid Research, New Jersey Institute for Food, Nutrition and Health, Rutgers University, New Brunswick, New Jersey, USA; d Department of Microbiology, Biochemistry and Molecular Genetics, New Jersey Medical School, Rutgers University, Newark, New Jersey, USA; Institut Pasteur

**Keywords:** *Cryptococcus neoformans*, phosphatidylserine synthase, PS synthase assay, inhibitor, bleomycin, mitochondria, ROS, antifungal drug

## Abstract

Invasive fungal infections are difficult to treat with limited drug options, mainly because fungi are eukaryotes and share many cellular mechanisms with the human host. Most current antifungal drugs are either fungistatic or highly toxic. Therefore, there is a critical need to identify important fungal specific drug targets for novel antifungal development. Numerous studies have shown the fungal phosphatidylserine (PS) biosynthetic pathway to be a potential target. It is synthesized from CDP-diacylglycerol and serine, and the fungal PS synthesis route is different from that in mammalian cells, in which preexisting phospholipids are utilized to produce PS in a base-exchange reaction. In this study, we utilized a Saccharomyces cerevisiae heterologous expression system to screen for inhibitors of Cryptococcus PS synthase Cho1, a fungi-specific enzyme essential for cell viability. We identified an anticancer compound, bleomycin, as a positive candidate that showed a phospholipid-dependent antifungal effect. Its inhibition on fungal growth can be restored by ethanolamine supplementation. Further exploration of the mechanism of action showed that bleomycin treatment damaged the mitochondrial membrane in yeast cells, leading to increased generation of reactive oxygen species (ROS), whereas supplementation with ethanolamine helped to rescue bleomycin-induced damage. Our results indicate that bleomycin does not specifically inhibit the PS synthase enzyme; however, it may affect phospholipid biosynthesis through disruption of mitochondrial function, namely, the synthesis of phosphatidylethanolamine (PE) and phosphatidylcholine (PC), which helps cells maintain membrane composition and functionality.

**IMPORTANCE** Invasive fungal pathogens cause significant morbidity and mortality, with over 1.5 million deaths annually. Because fungi are eukaryotes that share much of their cellular machinery with the host, our armamentarium of antifungal drugs is highly limited, with only three classes of antifungal drugs available. Drug toxicity and emerging resistance have limited their use. Hence, targeting fungi-specific enzymes that are important for fungal survival, growth, or virulence poses a strategy for novel antifungal development. In this study, we developed a heterologous expression system to screen for chemical compounds with activity against Cryptococcus phosphatidylserine synthase, Cho1, a fungi-specific enzyme that is essential for viability in C. neoformans. We confirmed the feasibility of this screen method and identified a previously unexplored role of the anticancer compound bleomycin in disrupting mitochondrial function and inhibiting phospholipid synthesis.

## INTRODUCTION

Opportunistic fungal infections remain a major cause of morbidity and mortality in immunocompromised patients. An estimated 1.5 to 2 million deaths worldwide every year are reported because of fungal infections, exceeding those killed by either malaria or tuberculosis (1). Among fungal pathogens, Cryptococcus neoformans, a common cause of fungal meningitis in immunocompromised patients, results in approximately 223,100 cases and 181,100 deaths per year globally (2). The treatment of fungal infections, including cryptococcosis, is limited to only three antifungal classes. Therefore, there is an urgent need to identify novel drug targets for the development of new antifungal drugs. However, these efforts are difficult because fungi and their mammalian hosts are both eukaryotes and many proteins that make potential targets for antifungal development are also conserved in humans.

Of the three classes of drugs (polyenes, azoles, and echinocandins) currently available to treat invasive fungal infections, most of them have therapeutic limitations. Amphotericin B, the best known fungicidal drug of class polyene that inhibits fungal ergosterol is associated with an array of systemic toxicities (3, 4). The combination of amphotericin B and flucytosine, which is the current standard treatment for cryptococcal meningitis (5), is not ideal due to the emergence of flucytosine resistance in C. neoformans and hepatotoxicity (6). Azoles, the most widely used class, are fungistatic, and their role in antifungal therapy is threatened by the emergence of azole resistance (7). Triazole resistance in Aspergillus fumigatus has been reported to significantly complicate the treatment of patients with aspergillosis, primarily affecting patients with a compromised immune system (8). Echinocandins, which target beta-1,3-glucan synthase, are fungicidal, but ineffective against C. neoformans. Additionally, there is a growing concern of echinocandin resistance in *Candida* spp (9). Recently, resistance to all classes of antifungal drugs has been characterized in human-pathogenic fungal species (10). These drawbacks of low efficacy, toxicity, and resistance which are associated with current available antifungals clearly necessitate the development of more effective and safer antifungal drugs.

**FIG 1:**
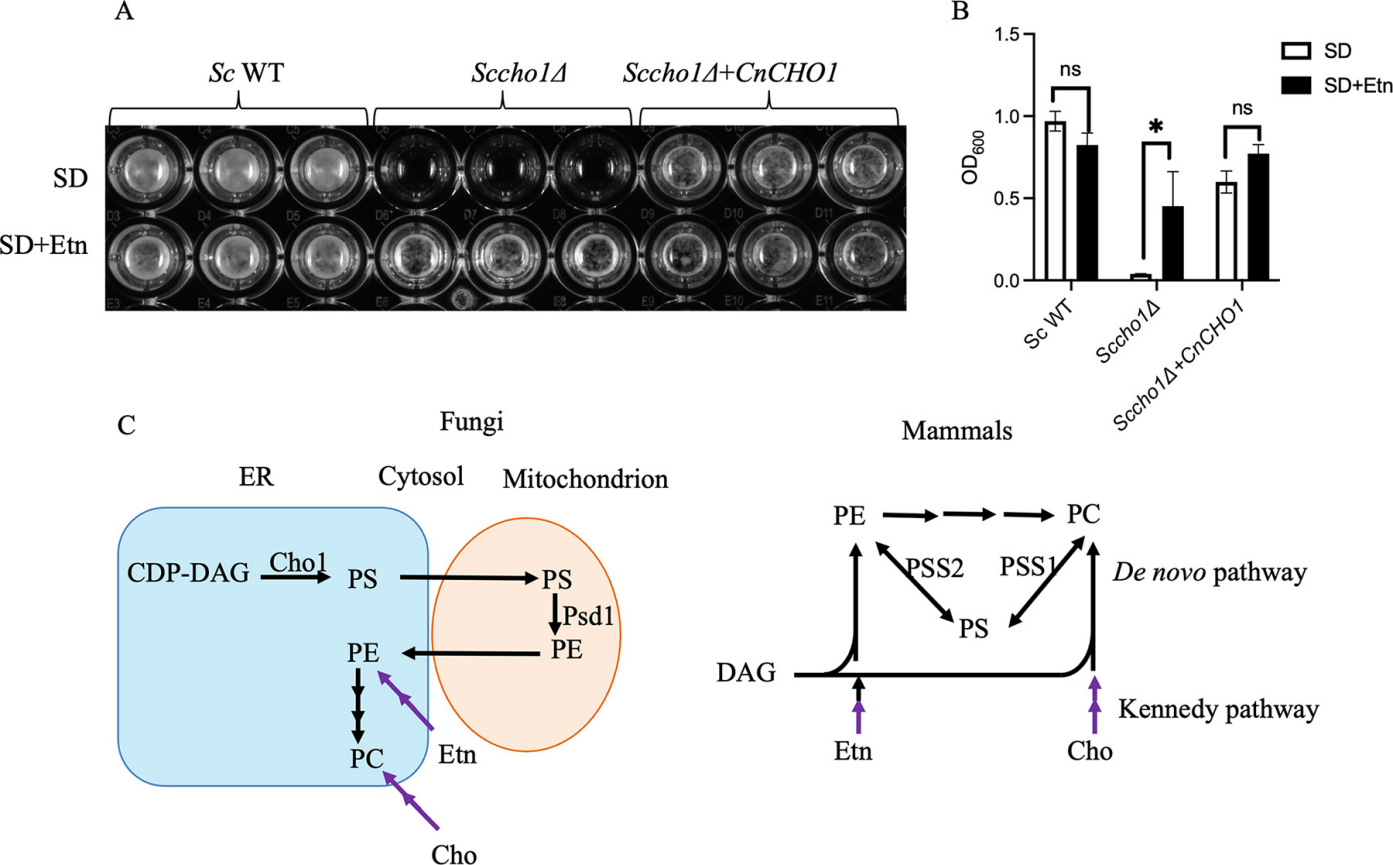
*CnCHO1* expression in *Sccho1*Δ mutant rescued its growth arrest phenotype. (A) Wild-type (WT) S. cerevisiae strain (*Sc* WT) grown in a 96-well plate at 30°C for 48 h showed no difference in growth on SD medium, both in presence and absence of ethanolamine. *Sccho1*Δ mutant failed to grow on SD medium lacking ethanolamine, while it grew on ethanolamine-supplemented medium. *Sccho1*Δ mutant expressing *CnCHO1* (*Sccho1Δ+CnCHO1*) grew well even in the absence of ethanolamine complementing the growth arrest. (B) Growth was evaluated via absorbance measurements at an optical density of 600 nm (OD_600_) using a plate reader. (C) PS synthesis in fungi and mammals. In yeast *de novo* pathway of phosphatidylserine (PS) synthesis, endoplasmic reticulum (ER)-localized PS synthase, and Cho1 converting CDP diacylglycerol (CDP-DAG) to PS. PS is subsequently converted to phosphatidylethanolamine (PE) by inner mitochondrial membrane-localized PS decarboxylase 1 (Psd1). PE can be exported to the ER for further conversion to phosphatidylcholine (PC). Ethanolamine (Etn) and choline (Cho) from cytosol can be utilized to form PE and PC, respectively, via the Kennedy pathway. In mammals, PS is produced through a base exchange reaction in which choline in PC and ethanolamine in PE are replaced with serine by the activity of PS synthases Pss1 and Pss2, respectively.

In fungi, phospholipids such as phosphatidylcholine (PC), phosphatidylethanolamine (PE), phosphatidylglycerol (PG), phosphatidylinositol (PI), and phosphatidylserine (PS) are major constituents of the fungal cell membrane, playing a variety of roles in cell specificity and pathogenicity (11). Several studies have reported the relevance of fungal phospholipid biosynthetic enzymes in the development of antifungal drugs. Due to substantial differences in the metabolic pathways in fungi and their mammalian hosts, one such enzyme, PS synthase (Cho1), has been proposed as a potential drug target (12–15). In fungi, PS is synthesized in the endoplasmic reticulum (ER) by PS synthase (Cho1) from two substrates, CDP diacylglycerol (CDP-DAG) and serine (13, 16). In mammals, PS is produced through a base exchange reaction in which the choline in PC and the ethanolamine in PE are replaced with serine by the activity of PS synthases Pss1 and Pss2, respectively (16) (Fig. 1). The conservation of Cho1 in fungi implicates a broad-spectrum antifungal application, and the absence of its homolog in mammals implicates a potential low-toxicity effect, if any, to human hosts, further indicating PS synthase inhibitor as a potential compound for antifungal development.

Disruption of PS synthase is known to impair varied functions in different fungi. In Saccharomyces cerevisiae, the *cho1*Δ mutant (*Sccho1*Δ) is viable but an ethanolamine/choline auxotroph (17). This mutant can only grow when supplemented with ethanolamine or choline, which is utilized to synthesize PE and PC via the Kennedy pathway (18). The homozygous *cho1*Δ/Δ mutant in C. albicans (*Cacho1*Δ/Δ) is viable and auxotrophic for ethanolamine (13), and its growth is very poorly rescued by choline supplementation. This mutant is avirulent in a murine model of systemic candidiasis, indicating the essentiality of *CaCho1* for virulence (13). The PS synthase mutant from fission yeast S. pombe (*pps1*Δ) is auxotrophic for ethanolamine and its growth defect cannot be rescued by choline (19). Unlike PS synthase in other fungi, Cho1 in C. neoformans (*CnCho1*) has been shown to be essential, and strains deficient in PS due to disruption of Cho1 cannot be rescued by ethanolamine or choline despite the presence of an active Kennedy pathway (20).

In this study, we established a yeast heterologous expression system by expressing C. neoformans *CHO1* in a S. cerevisiae *cho1Δ* mutant strain and utilized this system to screen compound collections for chemicals which specifically inhibit Cho1 function in C. neoformans. We screened a total of ~3,800 chemical compounds from three collections, namely, the Pathogen Box collection, the National Institutes of Health Clinical Collection (NCC), and the FDA-approved drug collection. Several compounds were identified based on their high potency and differential growth inhibition on synthetic defined (SD) medium in the presence and absence of ethanolamine. One of these is bleomycin, an anticancer agent commonly used in the treatment of multiple malignancies, including testicular cancer, head and neck cancer, cervical and uterine cancer, and pleural effusion caused by cancerous tumors (21). The mechanism of bleomycin-induced cytotoxicity is not entirely clear, but it likely includes oxidative damage that induces single- or double-stranded DNA breaks (22). This drug can disrupt mitochondrial function and induce the production of reactive oxygen species (ROS) (23). In our study, we explored the potential action of bleomycin in yeast cells and tested whether it can inhibit Cho1. Although our secondary screening yielded bleomycin as a promising hit, we found that this compound may not directly target Cho1. Because synthesized PS is translocated from the ER to the mitochondrial membrane to produce PE, our study demonstrated that bleomycin-induced mitochondrial dysfunction indirectly compromised phospholipid synthesis and homeostasis, e.g., PE and PC, leading to an inhibitory effect on fungal cells. In summary, our study identified the anticancer drug bleomycin as a potent antifungal target compound and revealed its potential mechanism in inhibiting fungal phospholipid synthesis.

## RESULTS

### Establishment of the yeast heterologous expression system for chemical screen of PS synthase.

Our previous study demonstrated that PS synthase Cho1 is an essential protein required for fungal viability and virulence in C. neoformans (20). In S. cerevisiae, the *cho1Δ* mutant (*Sccho1*Δ) is viable but ethanolamine-auxotrophic (17). When grown in SD medium, the *Sccho1*Δ mutant grew in the presence of 1 mM ethanolamine but failed to grow in its absence (Fig. 1A and B). This mutant was able to take up exogenous ethanolamine to synthesize PC and PE via the Kennedy pathway (Fig. 1C).

*Sccho1*Δ expressing the Cryptococcus *CHO1* gene (*CnCHO1*) was able to rescue its growth defect in ethanolamine-supplemented SD liquid medium. Therefore, we developed a screening assay based on this *Sccho1*Δ*+CnCHO1* complemented strain for functional screening of Cryptococcus PS synthase inhibitors. We predicted that *CnCho1* inhibitor would block growth of the *Sccho1*Δ+*CnCHO1* strain on medium lacking ethanolamine, mimicking an ethanolamine-auxotrophic phenotype.

### Primary screen identified compounds with inhibitory activity against *C. neoformans*.

Using the S. cerevisiae heterologous expression system (*Sccho1*Δ*+CnCHO1)*, we screened ~3,800 compounds from different collections for those which inhibit yeast growth in synthetically defined uracil dropout medium (SD-Ura). The primary objective of our screen was to identify not only potent antifungal compounds but also potential specific Cho1 enzyme inhibitors. We tested all 3 compound collections at a starting concentration of 5 μM and identified 46 compounds with MICs of 5 μM or lower from the FDA-approved drug collection. The Pathogen Box collection contained 6 compounds with MICs of 5 μM or lower, and NCC screening identified 7 compounds with MICs of 5 μM or lower. The preliminary screening yielded a diverse variety of compounds with various therapeutic uses like antihypertensive, antipsychotic, antihistamine, in addition to different known antifungal agents. The commercial use of each drug is listed together with their MIC values in Table 1. Some highly potent compounds such as thimerosal, temsirolimus, rapamycin, and phenylmercuric acetate were tested at lower concentrations. They exhibited effective antifungal activities against both S. cerevisiae and C. neoformans (MICs listed in Tables 1 and 2).

**TABLE 1:** Chemical compounds with MICs of ≤5 μM when tested against *Sccho1*Δ*+CnCHO1* strain^*a*^

Compounds and collection sources	Compound ID	MICs (μM)		Commercial use
		On SD	On SD+Etn	
FDA-approved				
Atorvastatin calcium		5	5	Cholesterol-lowering medication
Cetylpyridinium chloride		5	5	Antiseptic properties
Hexachlorophene		5	5	Disinfectant
Oxyquinoline sulfate		5	1.25	Antiseptic and disinfectant
Chloroxine		5	10	Antibacterial
Sirolimus		5	5	Immunosuppressant
Broxaldine		5	5	Antiprotozoal
Cetrimonium bromide		2.5	2.5	Antiseptic
Miltefosine		5	5	Anti-leishmanial
Carmofur		5	5	Antineoplastic
Broxyquinoline		5	5	Antiprotozoal
Crystal violet		5	2.5	Antibacterial, antifungal, and anthelminthic properties
Posaconazole		5	10	Antifungal drug
Chloroxine		5	5	Antibacterial
Sulconazole		5	2.5	Antifungal drug
Itraconazole		5	5	Antifungal drug
Miltefosine		5	5	Antimicrobial and anti-leishmanial
Clotrimazole		2.5	2.5	Antifungal drug
Gentian violet		2.5	2.5	Antibacterial, antifungal, and anthelminthic properties
Amorolfine hydrochloride		2.5	0.625	Antifungal drug
Nitroxoline		2.5	2.5	Antibacterial
Ebselen		2.5	2.5	Antioxidant activity
Disulfiram		2.5	1.25	Alcoholism medication
Econazole		2.5	1.25	Antifungal drug
Nitroxoline		2.5	1.25	Antineoplastic
Bleomycin		1.25	10	Antineoplastic
Econazole nitrate		1.25	0.312	Antifungal drug
Disulfiram		1.25	2.5	Alcoholism medication
Econazole nitrate		1.25	0.312	Antifungal drug
Caspofungin acetate		1.25	1.25	Antifungal drug
Fluorouracil		0.625	0.625	Antineoplastic
Oxiconazole nitrate		0.312	0.312	Antifungal drug
Tioconazole		0.625	0.625	Antifungal drug
Butoconazole		0.625	0.312	Antifungal drug
Sulconazole nitrate		0.625	0.625	Antifungal drug
Cycloheximide		0.625	0.625	Fungicide
Thiram		0.625	0.625	Fungicide
Climbazole		0.625	0.625	Antifungal drug
Bleomycin sulfate		0.625	10	Antineoplastic
Miconazole nitrate		0.312	0.312	Antifungal drug
Voriconazole		0.312	0.312	Antifungal
Pyrithione zinc		0.156	0.156	Fungistatic and bacteriostatic
Thimerosal		0.038	0.038	Antiseptic and antifungal agent
Temsirolimus		0.038	0.156	Antineoplastic
Rapamycin (sirolimus)		0.019	0.3125	Antineoplastic
Phenylmercuric acetate		0.0095	0.0095	Preservative and disinfectant properties
Pathogen Box				
NA	MMV688271	5	5	
NA	MMV102872	5	5	
Iodoquinol	MMV002817	2.5	2.5	Amebicidal
Posaconazole	MMV688774	0.625	0.625	Antifungal drug
NA	MMV687807	0.625	0.312	
Difenoconazole	MMV688943	0.625	0.625	Fungicide
NCC				
Hexachlorophene	SAM002554903	5	5	Antibacterial
Econazole nitrate	SAM002554898	2.5	5	Antifungal drug
Chloroxine	SAM002554895	1.25	1.25	Antibacterial
Miconazole nitrate	SAM002264623	1.25	1.25	Antifungal drug
Voriconazole	SAM001246664	0.625	0.625	Antifungal drug
Itraconazole	SAM001246679	0.625	0.625	Antifungal drug
Oxiconazole nitrate	SAM001246724	0.312	0.312	Antifungal drug

a SD, synthetic defined medium; SD+Etn, SD medium supplemented with 1 mM ethanolamine; NCC, NIH Clinical Collection.

**TABLE 2:** Highly potent compounds when tested against Cryptococcus WT strain H99^*a*^

Compounds	MIC (μM)
Temsirolimus	2.5
Rapamycin (sirolimus)	0.625
Pyrithione zinc	0.156
Thimerosal	0.078
Temsirolimus	0.038
Phenylmercuric acetate	0.0195

a WT, wild-type.

### Secondary screening of compounds that confer rescue in ethanolamine supplemented media.

**FIG 2:**
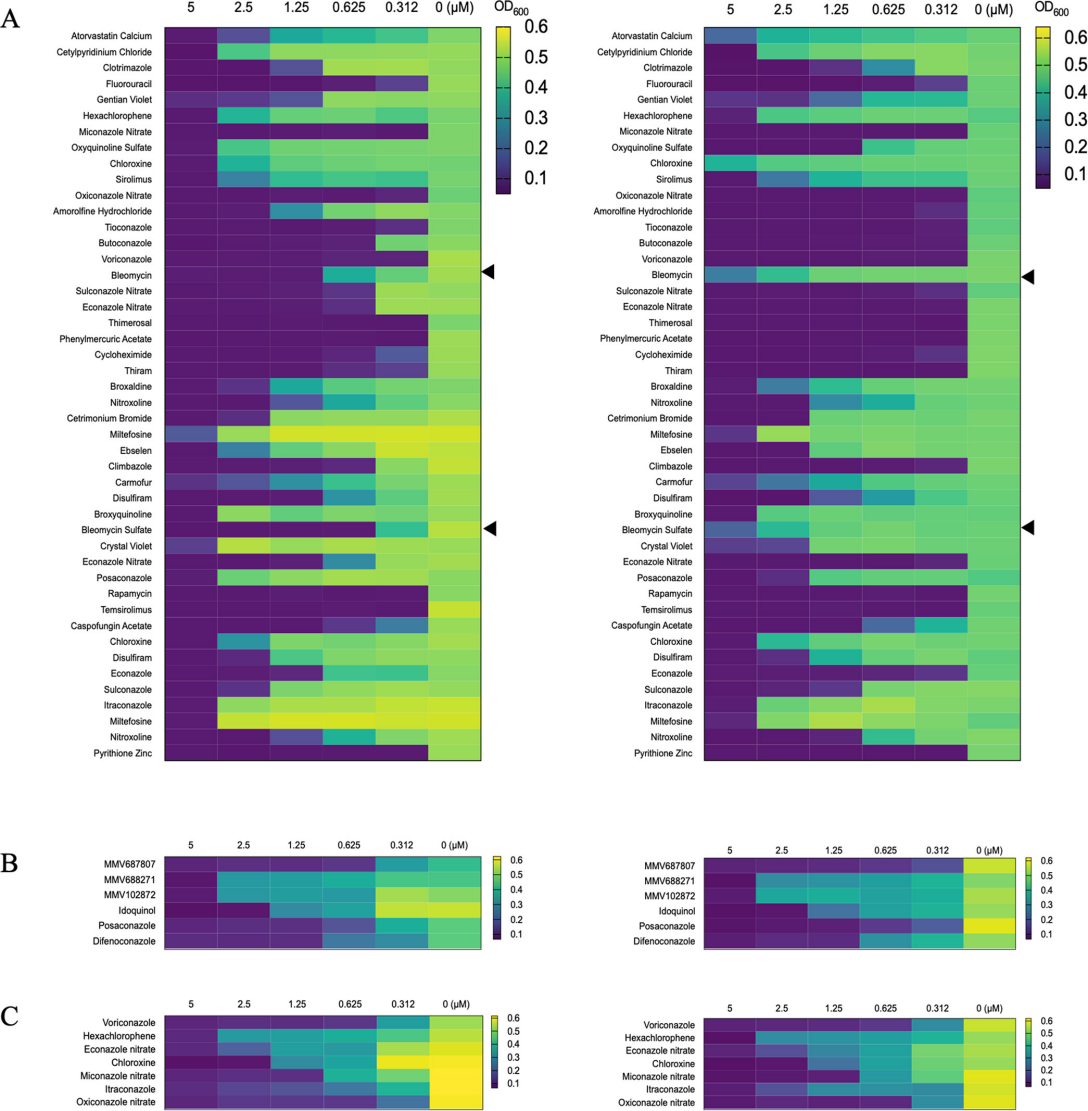
Secondary screening of compound collections identified compounds with some specificity against PS synthase. Primary screening at 5 μM starting concentration identified 46 chemical compounds in the FDA-approved drug collection (A), 6 compounds in the Pathogen Box collection (B), and 7 compounds in the NIH Clinical Collection (NCC) set (C). All chemical compounds from the primary screen were further examined for rescue of growth arrest in the presence of 1 mM ethanolamine (SD+Etn) compared to that in SD medium lacking ethanolamine (SD). Growth of *Sccho1*Δ*+CnCHO1* strain (1,000 cells) in 96-well plates in SD (left panel) and SD+Etn medium (right panel) at final concentrations of chemical compounds of 5, 2.5, 1.25, 0.625, 0.312, and 0 μM DMSO (dimethyl sulfoxide) was evaluated via absorbance measurements at OD_600_ using a plate reader after 48 h of incubation at 30°C. Results are represented as a heat map, with green indicating growth and purple indicating no growth. While most compounds showed similar ranges of growth inhibition, bleomycin and bleomycin sulfate (indicated by black arrowheads at the sides of the heat maps) showed some degree of growth rescue.

The secondary screening was based on rescue phenotypes in ethanolamine-containing medium, which indicated that the identified drug compounds targeted PS synthesis. We tested the effects of different concentrations of chemical compounds on their ability to rescue growth defect in SD medium supplemented with 1 mM ethanolamine (SD+Etn). Growth was evaluated via absorbance measurements at an optical density of 600 nm (OD_600_) using a plate reader, and the results were presented as a heat map (Fig. 2). For most of the possible drug targets, cell growth was not restored when growth medium was supplemented with 1 mM ethanolamine. Only bleomycin (plate 14 H09) and bleomycin sulfate (plate 21 C05) from the FDA-approved drug collection showed rescue phenotypes, as indicated by more than 5-fold increases in MICs in medium supplemented with ethanolamine compared to those in the medium lacking ethanolamine (Fig. 2A).

**FIG 3:**
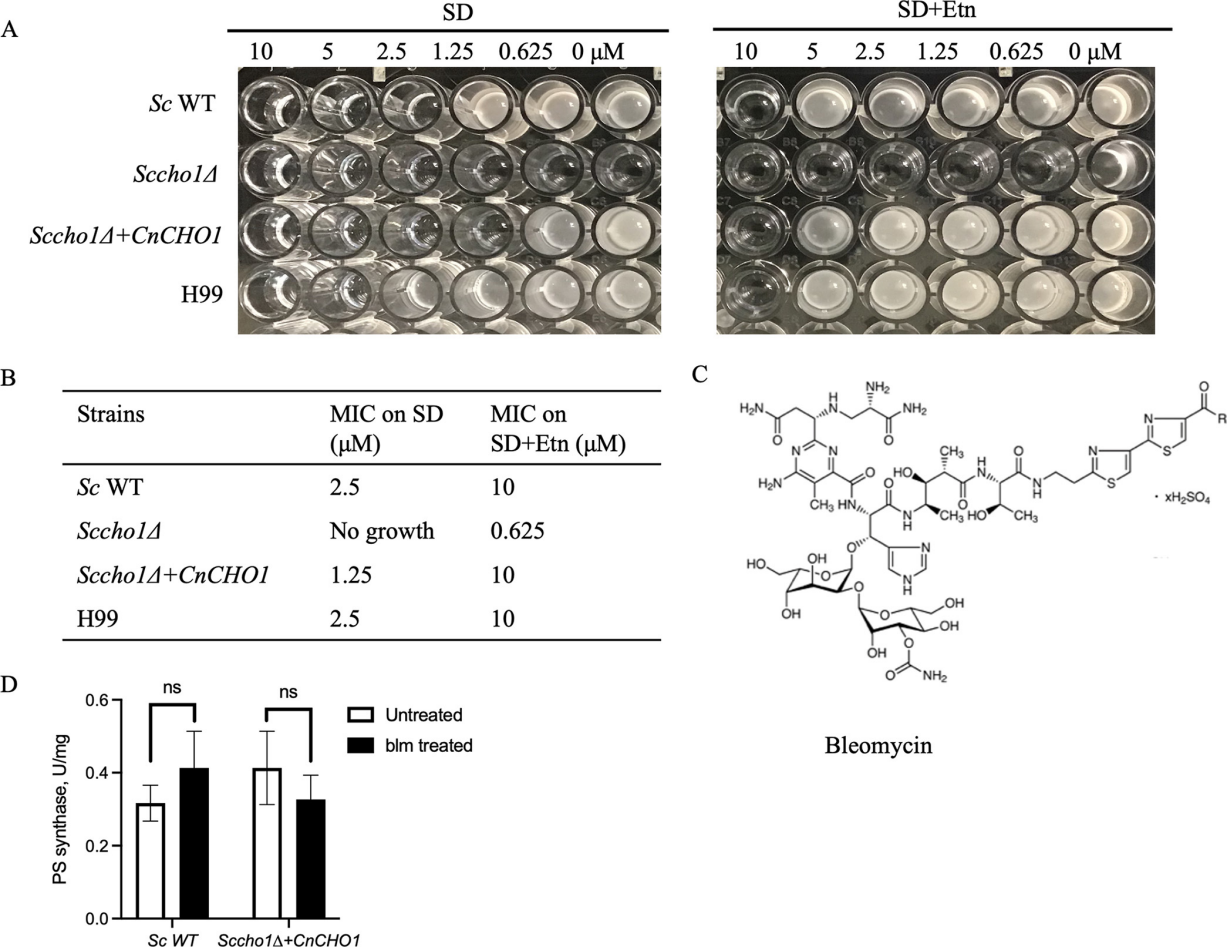
Determination of MICs and PS synthase activity. (A) MIC assay was performed on 96-well plate for indicated strains to determine the rescue effect of bleomycin (blm) in the presence of ethanolamine. (B) The lowest concentration that inhibited visible growth after 48 h of incubation is interpreted as the MIC. (C) Structure of bleomycin sulfate. (D) PS synthase activity in S. cerevisiae strains. Compared to the untreated condition, *Sc* WT and *Sccho1*Δ*+CnCHO1* with addition of 5 mM bleomycin showed no difference in PS synthase activity, indicating that bleomycin is not a specific inhibitor of Cho1. Data are represented as means ± standard deviation and statistical analysis was performed by unpaired two-tailed Student’s *t* test.

We tested bleomycin (purchased from TCI America, Portland, OR) activity against S. cerevisiae wild type (WT, YUX104), *Sccho1Δ* (YUX105), and *Sccho1Δ+CnCHO1* (YUX106) strains and C. neoformans wild-type strain (H99) in SD and SD+Etn media (Fig. 3A). The MICs of bleomycin against the *Sccho1*Δ*+CnCHO1* strain were 1.25 μM in SD medium and 10 μM in SD+Etn medium, while those H99 were 5 μM and 10 μM, indicating an 8-fold increase in MIC against *Sccho1*Δ*+CnCHO1* and a 2-fold increase in H99 in the ethanolamine-supplemented medium (SD+Etn), respectively (Fig. 3B). As expected, the *Sccho1*Δ mutant did not grow in the absence of ethanolamine and grew poorly in an ethanolamine-supplemented medium without bleomycin. The *Sc* WT strain showed 4-fold increases in MIC values (2.5 μM in SD and 10 μM in SD+Etn) (Fig. 3B). Thus, our data demonstrated that ethanolamine addition can rescue the antifungal activity of bleomycin in both S. cerevisiae and C. neoformans, suggesting that bleomycin may inhibit PS synthesis.

### Bleomycin does not inhibit PS synthase enzyme activity.

To determine whether bleomycin directly inhibits Cho1 enzyme activity, a PS synthase assay was performed for S. cerevisiae WT (YUX104) and *Sccho1*Δ*+CnCHO1* (YUX106) strains. In our previous study, we showed that the *Sccho1*Δ*+CnCHO1* strain restored PS synthesis activity in comparison to a *Sccho1*Δ mutant which completely lacked PS synthase activity (20). Based on that observation, we predicted reduced PS levels in the *Sccho1*Δ*+CnCHO1* strain under the bleomycin-treated condition if bleomycin specifically inhibits PS synthase enzyme, Cho1. Interestingly, we did not detect a significant difference in Cho1 enzyme activity for cells treated with 5 mM bleomycin compared to the untreated control in our direct PS synthase assay (Fig. 3D). These data show that bleomycin does not specifically inhibit the PS synthase enzyme.

### Bleomycin damages function of mitochondrial membrane.

**FIG 4:**
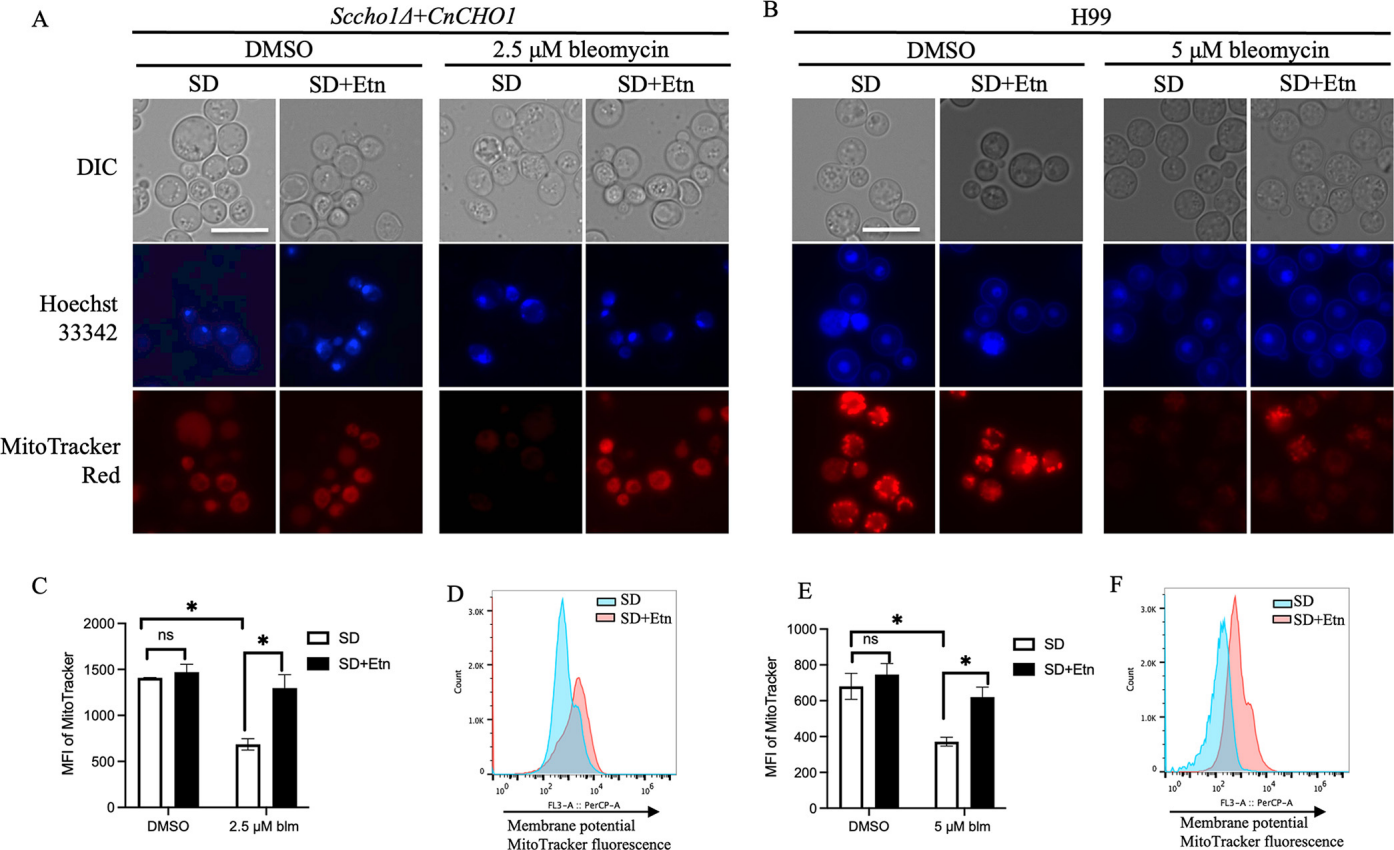
Analysis of mitochondrial function in bleomycin-treated yeast cells. The *Sccho1+CnCHO1* strain and C. neoformans WT (H99) cultured in SD and SD+Etn medium were treated for 10 h with 2.5 μM and 5 μM bleomycin, respectively. Cells stained with MitoTracker Deep Red FM and Hoechst 33342 were viewed under a florescence microscope. Under the bleomycin-treated condition, both *Sccho1+CnCHO1* (A) and H99 (B) showed accumulation of florescence dye in ethanolamine supplemented-medium, indicating normal mitochondrial membrane function. Scale bar = 10 μm. Flow cytometric analysis of MitoTracker-stained cells revealed higher mitochondrial membrane potential in yeast cells grown in ethanolamine-lacking medium. (C and E) Representative graphs of MitoTracker with flow cytometry for bleomycin-treated *Sccho1*Δ+*CnCHO1* (C) and H99 cells (E), respectively. (D and F) Overlaid fluorescence histogram of MitoTracker for cell populations from bleomycin-treated *Sccho1*Δ+*CnCHO1* (D) and H99 (F) cells. Values represent the average of three independent experiments, and error bars indicate standard deviation. Student’s *t* test was performed to determine statistical significance. *, *P* < 0.05; **, *P* < 0.01; ns, not significant.

Mitochondria are an important source of reactive oxygen species (ROS) (24, 25) which can lead to oxidative damage to mitochondrial proteins, membranes, and DNA, impairing its wide range of metabolic functions. Because PS synthesized in the ER needs to be translocated to the mitochondrial membrane to synthesize PC, we hypothesize that bleomycin inhibits fungal phospholipid production by targeting mitochondria function rather than by directly inhibiting Cho1 enzyme activity. To explore whether bleomycin targets mitochondrial function in C. neoformans, we used mitochondria-specific fluorescent dye MitoTracker Deep Red FM (Thermo Fisher Scientific, Waltham, MA) because its accumulation depends on mitochondrial membrane potential activity (21). After 10 h of treatment with bleomycin at a dose of 2× MIC (2.5 μM for S. cerevisiae and 5 μM for C. neoformans), mitochondrial function was monitored based on the retention of MitoTracker dye. Fluorescence imaging of MitoTracker probe exhibited a significant reduction of fluorescence intensity in ethanolamine-lacking, bleomycin-treated cells of both *Saccharomyces* and Cryptococcus spp. (Fig. 4A and B), indicating disrupted mitochondrial function. In contrast, bleomycin-treated cells grown in SD+Etn medium retained the dye, suggesting that the mitochondrial membrane was still intact and functional. All nuclei were visualized by Hoechst 33342 staining to determine intact cells and overall cell morphology. At the concentration used in our assay, there was no obvious difference in nuclear staining upon 10 h treatment with bleomycin. Furthermore, flow cytometry analysis revealed a reduced fluorescence signal intensity of MitoTracker dye in bleomycin-treated cells in the absence of ethanolamine, confirming the microscopic observations (Fig. 4C and E) for *Saccharomyces* and Cryptococcus yeast cells, respectively. Untreated yeast cells did not show a significant difference in the intensity of the MitoTracker signal when grown in SD medium with or without ethanolamine supplementation. An overlaid histogram of MitoTracker fluorescence intensity (blue, SD; red, SD+Etn) clearly demonstrates a loss of membrane potential in bleomycin-treated yeast cells in SD medium alone compared to that in ethanolamine-supplemented medium for both S. cerevisiae and C. neoformans (Fig. 4D and F). This indicates that bleomycin treatment led to perturbation of the mitochondrial membrane potential in SD medium, and that the membrane was protected to some extent in the medium supplemented with ethanolamine.

### Bleomycin induces ROS accumulation.

**FIG 5:**
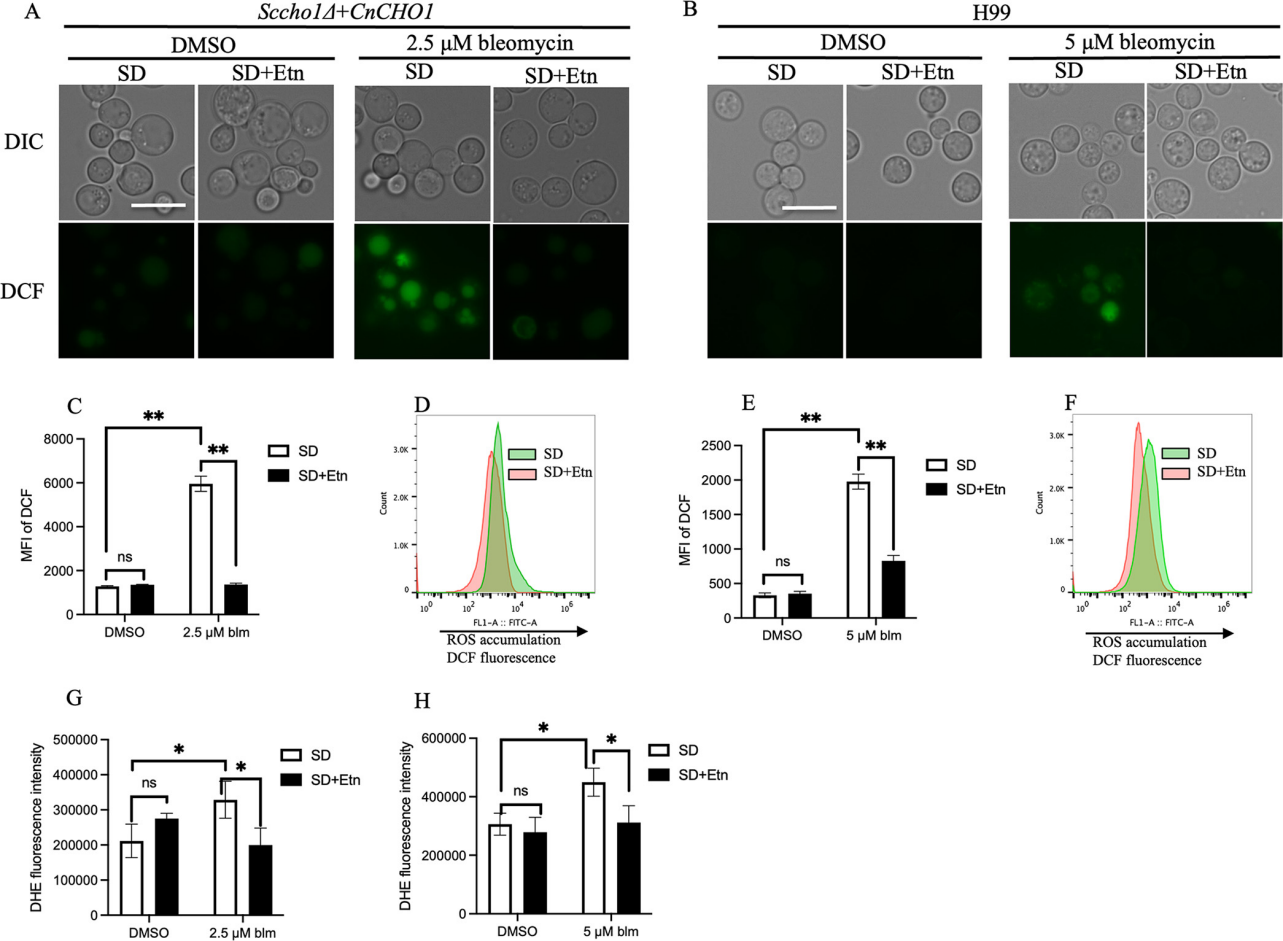
Effect of bleomycin treatment on reactive oxygen species (ROS) production in yeast cells. Overnight-incubated yeast cells were transferred to SD and SD+Etn media for 4 h before bleomycin treatment for 10 h. Intracellular ROS accumulation was assayed using 2′,7′-dichlorodihydrofluorescin diacetate (H_2_DCFDA) staining and visualized under a fluorescence microscope. Scale bar = 10 μm. Supplementation of ethanolamine decreased ROS accumulation in both strains, *Sccho1+CnCHO1* (A) and H99 (B). (C and E) Representative graph of dichlorofluorescein (DCF) with flow cytometry for bleomycin-treated *Sccho1*Δ+*CnCHO1* (C) and H99 (E) cells, respectively. (D and F) Overlaid fluorescence histogram of DCF for cell populations from bleomycin-treated *Sccho1*Δ+*CnCHO1* (D) and H99 (F) cells. (G and H) Intracellular ROS measurement using dihydroethidium (DHE). DHE fluorescence in *Sccho1*Δ+*CnCHO1* (G) and H99 (H) cells was analyzed using a microplate reader. Values represent the average of three independent experiments, error bars indicate standard deviation. Student’s *t* test was performed to determine statistical significance. *, *P* < 0.05; ns, not significant.

An impaired mitochondrial function increases the production of reactive oxygen species in yeast cells (26). Furthermore, independent of mitochondrial membrane potential damage, bleomycin itself is also known to cause an increase in ROS, resulting in oxidative stress and apoptosis (27). Therefore, we measured ROS levels in yeast cells to determine whether bleomycin treatment contributes to increased ROS accumulation and whether it varied when grown in ethanolamine-supplemented SD medium. Yeast cells grown overnight in SD medium were transferred to SD and SD+Etn medium for an additional 4 h followed by 10 h treatment with bleomycin. Intracellular ROS accumulation was measured using fluorescent dyes, 2′,7′-dichlorodihydrofluorescein diacetate (H_2_DCFDA) and dihydroethidium (DHE). The readout of both probes is a fluorescence signal that enables the measurement of ROS production in fungal cells. The H_2_DCFDA assay is based on intracellular esterase action on non-fluorescent H_2_DCFDA to release an intermediate product which reacts with ROS to form the fluorescent product 2′,7′-dichlorofluorescein (DCF) (28). Fluorescence imaging of H_2_DCFDA probe demonstrated a significant increase in ROS production in cells grown in SD with bleomycin compared to that in SD+Etn for both S. cerevisiae and C. neoformans yeast cells (Fig. 5A and B), respectively. Concurrent with microscopic data, flow cytometry analysis revealed increased fluorescence intensity of the H_2_DCFDA probe in ethanolamine-lacking, bleomycin-treated cells of both *Saccharomyces* and Cryptococcus spp. (Fig. 5C and D). Untreated yeast cells showed no significant difference in the intensity of ROS probe staining when grown in medium with or without ethanolamine. A histogram of ROS fluorescence intensity (green, SD; red SD+Etn) clearly exhibited increased ROS accumulation in bleomycin-treated yeast cells in SD medium alone compared to that in ethanolamine-supplemented medium in both yeast species (Fig. 5D and F). This observation suggests that ethanolamine in the medium protected the yeast cells from bleomycin-induced damage.

To confirm the findings from H_2_DCFDA probe data, we employed another fluorescent probe, DHE, to measure ROS production. Fluorescence signal resulting from the oxidation of DHE to 2-hydroxyethidium by superoxide has been used as an assay to measure ROS. The same procedure for yeast cell preparation was followed for the DHE assay, except that we utilized a microplate reader to detect fluorescence signal. Similar to the previous results from the H_2_DCFDA assay, bleomycin-treated yeast cells in SD medium showed increased ROS accumulation compared to those grown in ethanolamine-supplemented medium for both *Saccharomyces* and Cryptococcus spp. (Fig. 5G and F). The increased ROS accumulation by both methods upon treatment with bleomycin in SD medium lacking ethanolamine demonstrates increased the susceptibility of yeast cells to bleomycin-induced damage.

## DISCUSSION

There is an urgent public health need for novel, potent antifungal drugs due to the increase in life-threatening fungal infections. This study was designed to screen for compounds with antifungal activity against PS synthase, which represents a new fungal target because it is fungi-specific and essential for Cryptococcus viability. To date, no direct inhibitor of fungal PS synthase enzymes has been identified to have antifungal drug potential. Previous screens for inhibitors of PS synthase (Cho1) in *Candida* spp. identified the small molecule SB-224289, a serotonin receptor antagonist, as a positive hit; however, a PS synthase assay displayed no change in the activity of Cho1 (29). In our study, we developed a whole-cell functional screen strategy using a yeast heterologous expression system to screen for inhibitors of Cryptococcus PS synthase. Of all those which displayed antifungal properties, bleomycin, an anticancer agent, was identified as a promising candidate due to its low MICs against both S. cerevisiae and C. neoformans and its ability to restore growth inhibition by the addition of ethanolamine. Our study, hence, focuses on the mechanism of bleomycin inhibition of C. neoformans.

Bleomycin has been clinically used as a cancer chemotherapeutic agent for the treatment of several types of malignancies; however, its mechanism of action remains unclear and is limited mostly to cancer cells. The cytotoxic action of bleomycin has been attributed to an activated form that induces sequence-specific single- and double-stranded DNA breaks (30), RNA degradation (31, 32), and lipid peroxidation (33). The potential antifungal activity of bleomycin has been explored in some fungi. It has been reported to have MICs of 6.4 μg/mL in C. neoformans, 0.39 to 12.5 μg/mL in *Candida* spp., and 3.2 μg/mL in S. cerevisiae (34). In another study, bleomycin showed an MIC of 3.2 μg/mL against Aspergillus fumigatus, with inhibition of conidial germination and hyphal development and a defect in cell wall septation (35). The direct target of bleomycin on fungal cells such as C. neoformans remains unknown.

To determine whether bleomycin directly inhibits PS synthase Cho1 function, we performed a PS synthase enzyme activity assay. Interestingly, no significant difference in Cho1 enzyme activity was detected in the presence of bleomycin treatment, suggesting that bleomycin may not specifically inhibit Cho1 function to block PS synthesis. This result explains our MIC assay data, in which not only the *Sccho1*Δ*+CnCHO1* strain but also *Sc* WT and *Cn* WT showed exogeneous ethanolamine-dependent MIC changes. While *Sccho1*Δ*+CnCHO1* was demonstrated to be an ethanolamine auxotroph, *Sc* WT and *Cn* WT also showed some degree of growth rescue in the presence of ethanolamine (Fig. 3A).

Because reports have shown that some anticancer drugs target mitochondrial function to induce apoptosis (36), and PS synthesized in the ER is translocated to the mitochondrial membrane to produce PE through PS decarboxylase, we assessed bleomycin’s potential to inhibit mitochondrial function. In our study, we observed reduced mitochondrial membrane potential in bleomycin-treated yeast cells grown in SD medium, but not in cells grown in ethanolamine-supplemented medium (Fig. 4). As with the mitochondrial-staining results, the ROS assay also indicated greater ROS accumulation in bleomycin-treated yeast cells grown in SD medium than in cells grown in SD medium supplemented with ethanolamine (Fig. 5). Increased ROS production in yeast cells grown in SD medium could be an independent effect of bleomycin or the result of mitochondrial disruption. Taken together, both the mitochondrial staining and ROS assays indicate bleomycin-induced damage in yeast cells grown in SD medium and that cells are somewhat protected in SD+Etn medium. We hypothesize that bleomycin-induced inhibition in yeast cells could be the result of impaired mitochondrial function which possibly disrupts enzymes located in the mitochondrial membrane, including PS decarboxylase Psd1, which synthesizes PE from PS. Studies have shown that cardiolipin (CL) and PE are essential phospholipids for mitochondrial respiratory function (37), and that their absence or reduced levels are known to cause serious growth defects in cells (38). Our observation showed that exogeneous supplementation of ethanolamine helped yeast cells preserve membrane functionality without drastically affecting the cells upon bleomycin treatment. In contrast, in the absence of ethanolamine, mitochondrial impairment induced by bleomycin treatment drastically decreased levels of phospholipids (e.g., PE) inside the cells, making them more susceptible to damage. It is clear that in S. cerevisiae, the Kennedy pathway plays a critical role in PE and PC synthesis when enzymes of the *de novo* pathway are disrupted (39). Enabling PE and PC synthesis via the Kennedy pathway, the Sc*cho1*Δ mutant can grow if ethanolamine or choline are exogenously supplied (40). We believe that bleomycin treatment damaged the mitochondria, where phospholipid biosynthesis enzymes localize, consequently disrupting one or many key pathway enzymes. The supplementation of ethanolamine, however, activated the ER-localized Kennedy pathway, which bypasses the mitochondria to generate PE and PC to support biogenesis and maintain cell viability. This hypothesis will be tested in future studies.

In addition to identifying bleomycin and its phospholipid-dependent antifungal effect, our screen also led to the identification of an extensive list of known antifungal and non-antifungal compounds against S. cerevisiae and C. neoformans (Table 1). Our screen of the Pathogen Box collection identified several antifungal drugs which were also identified in a previous study of C. neoformans and C. albicans, e.g., difenoconazole, posaconazole, MMV688271 (41), and MMV687807 in C. albicans (42). Sirolimus or rapamycin, initially discovered as an antifungal agent (43) and then as an immunosuppressive drug, was shown to have robust antifungal activity against both S. cerevisiae (MIC = 0.019 μM) and C. neoformans (MIC = 0.625 μM). Similar studies in both C. albicans and S. cerevisiae showed inhibition by low concentrations of rapamycin (MIC = <0.0625 μg/mL) (44). Some potent antifungal compounds such as pyrithione zinc (MIC = 0.156 μM), thimerosal (MIC = 0.078 μM), temsirolimus (MIC = 0.038 μM), and phenylmercuric acetate (MIC = 0.0195 μM) were also identified. However, we did not further investigate them because they are being used as antifungals. The antifungal properties of zinc pyrithione (45), widely used in anti-dandruff treatment, and mercury-based compounds such as thimerosal (46) and phenylmercuric acetate (47), which are effective against ocular-pathogenic fungi, are used in ophthalmic medication.

In summary, we found that bleomycin exhibits potent antifungal activity and that the concentration of available ethanolamine in the medium changes the effect of bleomycin treatment in yeast cells. Our data indicate that bleomycin inhibition of fungal growth may not be due to directly targeting PS synthase Cho1, but rather the phospholipid synthesis pathway downstream of PS production. The mechanism of Cho1 involvement in this process remains to be explored. Further studies are warranted to better understand bleomycin’s mode of action in the phospholipid *de novo* biosynthetic pathway and the interplay between the biosynthetic and Kennedy pathways in fungal cells.

## MATERIALS AND METHODS

### Yeast strains and media.

All strains used in this study are listed in Table 3. Fungal strains were grown in synthetic dropout medium lacking uracil (SD-Ura) (0.67%, yeast nitrogen base without amino acids, 2% glucose with appropriate supplement of 1× all amino acids except uracil), except for strain YUX105 which was maintained in SD-Ura supplemented with ethanolamine. All compound screening assays were performed on SD-Ura medium with or without 1 mM ethanolamine (SD+Etn and SD, respectively). Overnight cultures were grown in liquid SD-Ura medium in a shaking incubator at 30°C and 200 rpm.

**TABLE 3:** Strains used in this study^*a*^

Strains	Genotype	References
YUX104	*MAT*a *ade2-1 can1-100 his3-11,15 leu2-3,112 trp1-1 ura3-1* [pTH19] (Saccharomyces cerevisiae WT)	20
YUX105	*MAT*a *ade2-1 can1-100 his3-11,15 leu2-3,112 trp1-1 ura3-1 cho1*Δ::*TRP1* [pTH19] (*Sccho1*)	20
YUX106	*MAT*a *ade2-1 can1-100 his3-11,15 leu2-3,112 trp1-1 ura3-1 cho1*Δ::*TRP1* [pTH19-*Cn CHO1*] (*Sccho1+CnCHO1*)	20
H99	*MAT*α Cryptococcus neoformans WT	52

a WT, wild-type.

### Drug library and drug reconstitution.

The Pathogen Box collection (*n* = 400) was kindly provided by the Medicines for Malaria Venture (MMV) (https://www.mmv.org) with a stock concentration of 10 mM/10 μL compound in each well in dimethyl sulfoxide (DMSO). The National Institutes of Health Clinical Compound Collection (NCC) (*n* = 707) was provided by the NIH Small Molecule Repository (https://pubchem.ncbi.nlm.nih.gov/source/NIH%20Clinical%20Collection) with a stock concentration of 10 mM/10 μL compound in each well. Compounds from both collections were dissolved in filter-sterilized DMSO (Sigma-Aldrich, St. Louis, MO) and diluted in sterile double-distilled water (ddH_2_O) to make stock solutions of 10 μM/μL. The FDA-approved drug collection (*n* = 2662) was provided by Molecular Design and Synthesis Core, Rutgers University with concentrations of 5,000 pmol compound in each well. The dried-out compounds were completely dissolved in 10 μL DMSO and diluted in sterile ddH_2_O to make working solutions of 50 μM compound. All stock chemical compounds in a master plate were stored at −80°C. A large quantity of bleomycin sulfate (mixture) was purchased from TCI America (Thermo Fisher Scientific) and prepared at 10 mM using DMSO as the solvent.

### Primary screening of drug library.

Screening of the compounds was conducted in a sterile polystyrene, flat-bottomed 96-well plate (Corning). Overnight cultures in SD-Ura medium were washed twice in sterile phosphate-buffered saline (PBS; 137 mM NaCl, 2.7 mM KCl, 10 mM Na_2_HPO_4_, 1.8 mM KH_2_PO_4_) resuspended in fresh SD medium, and incubated for at least 2 h to make fresh yeast suspension. A total of 1,000 yeast cells, as measured by hemocytometer, were exposed to identical starting concentrations of 5 μM drug for each library, to a final volume of 100 μL SD medium, and incubated without agitation at 30°C. Chemical compounds which visibly inhibited yeast cell growth after 48 h were recorded and further assessed into secondary screening. In the primary screen, highly effective compounds were tested again at lower MICs.

### Secondary screening for antifungal agent targeting PS synthesis and assessment of MICs.

Here, 96-well plates were set out with SD medium in one half (6 wells) and SD medium with 1 mM ethanolamine in the other half (6 wells). Reconstituted drug from the master plate was transferred to the first well to serially dilute the drug to half-concentration to obtain final concentrations of 5, 2.5, 1.25, 0.625, and 0.312 μM and the control (without drug, DMSO vehicle only) to a final volume of 100 μL. A total of 1,000 yeast cells were inoculated in each well and plates were incubated without agitation at 30°C. After 48 h of incubation, OD_600_ measurements were performed using a Perkin Elmer EnVision2104 Multilabel Reader and visualized quantitatively by a heatmap using Prism version 8.0 (GraphPad Software, Inc.). Assays for bleomycin MICs were performed in sterile 96-well plates as described above, but starting from higher drug concentrations to determine the MIC in SD+Etn medium.

### PS synthase activity assay.

PS synthase assays were performed for *Sc* WT and *Sccho1*Δ*+CnCHO1* strains. Cells were grown at 30°C in SC-Ura medium to the exponential phase (OD_600_ ≈ 0.6) and harvested at 1,500 × *g* for 10 min. The harvested cells were resuspended in breaking buffer (50 mM Tris-HCl [pH 7.5], 0.3 M sucrose, 10 mM 2-mercaptoethanol, and Roche EDTA-free protease inhibitor cocktail), mixed with glass beads (0.5-mm diameter), and lysed using a Mini-BeadBeater-16 (BioSpec Products, Bartlesville, OK). The cell lysate was centrifuged at 1,500 × *g* for 10 min at 4°C, and the supernatant was used as cell extracts. PS synthase activity was measured at 30°C for 20 min in 100 μL reaction mixture containing 50 mM Tris-HCl (pH 8.0), 0.6 mM MnCl_2_, 0.2 mM CDP-DAG, 4 mM Triton X-100, 0.5 mM (3-^3^H)serine (10,000 cpm/nmol), and 10 μg cell extracts (48). The effect of bleomycin was examined at a final concentration of 5 mM. A unit of PS synthase activity was defined as the amount of enzyme that catalyzed the formation of 1 nmol product/min per mg of protein (U = nmol/min/mg protein). The enzyme assays were performed in triplicate and repeated twice.

### Mitochondria and DNA staining, fluorescence microscopy, and flow cytometry.

Overnight yeast cells grown in 3 mL liquid SD medium at 30°C with constant agitation were collected at 3,000 × *g* for 2 min the next morning and transferred in SD and SD+Etn medium for 2 to 4 h. The initial cell density was adjusted to around OD_600_ = 0.3 before treatment with bleomycin (2.5 μM for *Sc* and 5 μM for H99) for 10 h. Cells were harvested, washed once in PBS, and resuspended in PBS supplemented with 0.2 μM MitoTracker Deep Red FM (Invitrogen) and 1 μg of Hoechst 33342 (Thermo Fisher Scientific). Cells were incubated for 10 min at 30°C in the dark, washed twice with PBS, resuspended in PBS, and observed under a fluorescence microscope (Olympus BX61, Olympus Life Sciences). MitoTracker Deep Red fluorescence was detected by flow cytometry with a BD Via-Probe (BD Biosciences) using FL3 channel. The data were analyzed using FlowJo software (FlowJo LLC) and expressed as mean fluorescence intensity (MFI). All experiments were done in triplicate. Analysis was performed using the unpaired, two-tailed *t* test in GraphPad Prism version 8. *P* values of <0.05 were considered statistically significant.

### Measurement of ROS.

ROS production was detected using dichlorodihydrofluorescein diacetate by either flow cytometry or under a fluorescence microscope as previously reported (49, 50) but with slight modifications. Cells were grown overnight in 3 mL SD-Ura. The next day, cells were centrifuged at 3,000 × *g* for 2 min and incubated in fresh SD and SD+Etn medium for few hours until the cell density (OD_600_) reached around 0.3. Cells were treated with either 0.1% (vol/vol) DMSO (vehicle control) or bleomycin (2.5 μM for *Sc* and 5 μM for H99) and incubated at 30°C with constant agitation for 10 h. Cells were harvested from the culture medium and centrifuged at 3,000 × *g* for 2 min and washed with PBS. To detect ROS, cells were resuspended in PBS supplemented with H_2_DCFDA (Invitrogen) (2.5 μM for *Sc* and 5 μM for H99) and incubated in the dark at 30°C with constant agitation for 30 min. Cells were washed twice with PBS to remove excess dye and resuspended in PBS. Fluorescence signal was immediately detected by flow cytometry with BD Via based on FL1 channel to detect ROS. For microscopic analysis, cells were visualized at 100× magnification using a fluorescence microscope (Olympus BX61). Intracellular ROS levels were also measured with dihydroethidium (DHE, Invitrogen) as described previously in yeast cells, with some modifications (51). For the DHE assay, yeast cells were prepared in the same way. After treatment with bleomycin for 10 h, washed yeast cells were incubated with DHE (10 μM for *Sc* and 20 μM for H99) for 1 h. Cells were washed twice with water and 200-μL samples were added to each well of a 96-well microplate. A control set of samples incubated without fluorescent dye were included as unstained cells. ROS was measured as fluorescence emitted by the fluorescent dye using a SpectraMax iD5 Microplate Reader (Molecular Devices, San Jose, CA) with excitation and emission wavelengths of 518 and 606 nm, respectively. The obtained reading was normalized against the unstained cells and data were expressed as DHE fluorescence intensity. All experiments were done in triplicate. Analysis was performed using the unpaired, two-tailed *t* test in GraphPad Prism version 8. *P* values of <0.05 were considered statistically significant.
